# Reduction of Salt and Fat in Frankfurter Sausages by Addition of *Agaricus bisporus* and *Pleurotus ostreatus* Flour

**DOI:** 10.3390/foods9060760

**Published:** 2020-06-09

**Authors:** Magdalena I. Cerón-Guevara, Esmeralda Rangel-Vargas, José M. Lorenzo, Roberto Bermúdez, Mirian Pateiro, Jose A. Rodríguez, Irais Sánchez-Ortega, Eva M. Santos

**Affiliations:** 1 Universidad Autónoma del Estado de Hidalgo, Área Académica de Química, Crta. Pachuca-Tulancingo Km 4.5 s/n, Col. Carboneras, Mineral de la Reforma, HID 42183, Mexico; magdacerondcash@gmail.com (M.I.C.-G.); esmeralda_rangel10403@uaeh.edu.mx (E.R.-V.); josear@uaeh.edu.mx (J.A.R.); irais_sanchez5498@uaeh.edu.mx (I.S.-O.); 2 Meat Technology Centre of Galicia, Rúa Galicia Nº 4, Parque Tecnológico de Galicia, San Cibrao das Viñas, 32900 Ourense, Spain; jmlorenzo@ceteca.net (J.M.L.); robertobermudez@ceteca.net (R.B.); mirianpateiro@ceteca.net (M.P.)

**Keywords:** edible mushroom flour, *Agaricus bisporus*, *Pleurotus ostreatus*, healthier meat products

## Abstract

The reduction of fat and salt and the incorporation of fiber-rich compounds in frankfurters is a trend to improve their nutritional profile. The objective of this study was to evaluate the partial replacement of 30 and 50% of pork backfat and 50% of salt by adding edible mushroom flour (2.5 and 5%) from *Agaricus bisporus* (Ab) and *Pleurotus ostreatus* (Po) on physicochemical, microbiological and sensory properties of frankfurters sausages during cold storage. The addition of flours increased the moisture, and the dietary fiber contents in frankfurters, keeping the amino acid profile. Lipid oxidation remained under acceptable values despite not antioxidant effect was observed by mushrooms flours. Only spore-forming bacteria were found during cold storage. Color and texture was modified by addition of mushroom, being the Ab samples darker, while Po flour addition resulted in softer and less cohesive sausages. Although lower color, flavor, and taste scores were given to the mushroom samples than the control, they ranked in the acceptable level confirming that the inclusion of 2.5 and 5% of Ab and Po flours in fat- and salt-reduced frankfurter sausages resulted a feasible strategy to enhance the nutritional profile these products.

## 1. Introduction

Meat and meat products have excellent nutritional properties, being sources of protein, fat, essential amino acids, minerals and vitamins [1,2]. However, due to its higher lipid profile and sodium content, apart from other contaminants present after obtaining and processing, meat consumption has lately been connected to the development of different diseases around the world, mainly diabetes, obesity, cardiovascular problems and cancer [3]. Therefore, the increasing demand for healthier meat products has driven to the meat industry and scientific community to look for new meat products with less fat and salt content, with dietary fiber addition, and even probiotics inclusion, or natural antioxidants and vegetable proteins addition [4,5,6].

The reduction of fat or salt and substitution by other vegetable sources or ingredients to improve the functional and nutritional properties has been widely tested in emulsified sausages like frankfurters considering these sausages may contain up to 30% pork fat, 40% of which is saturated fatty acids [7,8]. However, since they are highly demanded and consumed, maintaining the sensory quality in the improvement of nutritional value is essential [9].

In this sense, different oil sources like olive, canola, linseed and fish oil as well as cereal and seed fibers (rice, chia, linseed) or vegetables, legumes and fruits, even algae, have been included in frankfurters considering the nutritional properties but also the technological issues associated to the emulsion formation [10,11,12,13,14,15,16]. One alternative to improve the nutritional profile of meat products could be the edible mushrooms since they have been reported to be a rich source of essential nutrients, with a high content in protein (16.47–36.96%), low level of lipids, and high dietary fiber content (24.4–46.62% in dry weight, DW) [17]. Because of their interesting flavor, medicinal and quality nutritional aspects, they have been used as food supplement to prevent malnutrition [18]. Apart from that, the strong umami taste of mushroom often referred to as a meaty taste [19,20] could contribute to reduce the salt content in meat formulations [21].

Moreover, cultivation of edible mushrooms is an easily and economically viable process, due to their ability to grow on different wastes such as cereal, cotton, fruit, vegetable, sawdust and leaf [22]. Several successful attempts to use mushroom flour in some bakery products have been reported [23,24,25] but less information is available about its incorporation in meat products with different results [8,26]. Due to the above reasons, mushrooms could be considered a promising naturally functional ingredient to improve nutritional quality of meat products, being *Agaricus bisporus* (Ab; champignon) and *Pleurotus ostreatus* (Po; oyster mushroom) the most worldwide cultivated species [27]. The aim of this investigation was evaluate the effect of partial replacement of fat and salt by addition of edible mushroom (Ab and Po) flour in frankfurter type sausages on the physicochemical, microbiological and sensorial parameters during cold storage.

## 2. Materials and Methods

### 2.1. Flour Preparation

Lots of 4 kg from Ab and Po mushroom species were purchased from a local market in Mineral de la Reforma (Hidalgo, Mexico). Mushrooms were carefully selected based on visual appearance (light color without visible damage) and then rinsed, drained and cut in 5 mm-thick slices. Pieces of Ab were immersed in an acetic acid solution (2%) at 80 °C for 10 min and water-cooled at 4 °C to prevent enzymatic reactions. After draining off the excess liquid, the samples were dried in air-recirculating oven (CE5F, Shel Lab, Cornelius, OR, USA) at 60 ± 3 °C for 18 h, milled in a UDY cyclone sample mill (UDY Corp., Fort Collins, CO, USA) and sleeved through a 0.5 mm mesh. The resultant flours were placed in hermetic polyethylene bags and stored in the dark at room temperature until use.

### 2.2. Manufacture of Frankfurter-Type Sausages

Frankfurters were elaborated in the Meat Technology Centre (Ourense, Spain). Five formulations were designed to reduce fat (30% and 50% reductions) and salt, phosphates and caseinate (50% reduction) by the addition of Ab and Po flours (2.5% and 5.0%), comparing with the control formulation (C) elaborated with 25% fat and 1.5% salt, 2% of sodium caseinate and 0.5% of phosphates (Table 1). MF-Ab and MF-Po were the codes to identify sausages with 30% of fat reduction (medium-fat) and 50% of salt-phosphates-caseinate reduction, and with 2.5% Ab and Po flours, respectively. LF-Ab and LF-Po were the codes used for the sausages with 50% fat reduction (low-fat) and 50% salt-phosphates-caseinate reduction, and 5.0% Ab and Po flour added, respectively. MF-PoAb were assigned to identify the sausages with 30% fat reduction and 50% salt-phosphates-caseinate reduction, incorporated with 5.0% Ab and Po mix flour (2.5% each one).

Before the manufacturing process, sodium chloride and sodium ascorbate were added to the meat cut in 1 cm cubes with a rest period of 2 h. The lean and fat were minced, using plates of 8 mm and 6 mm respectively in a refrigerated mincer machine (La Minerva, Bologna, Italy). Potato starch and 50% of total sodium caseinate (dissolved in water) were previously added to the meat. Then, the rest of ingredients with the fat were added and mixed to homogeneity in a chilled cutter (TALSA K30 0542, Talsabell S.A., Valencia, Spain). Meat batter was stuffed into 25 mm collagen casings with an automatic equipment (SIA, Junior, Barcelona, Spain) forming 10 cm frankfurters.

The raw sausages were cooked in a temperature-controlled bath-water (Marmite Mera REA-505, Talsa, Talsabell S.A., Valencia, Spain) at 90 °C for 20 min. Then, the cooked sausages were cooled in an ice water-bath. Four frankfurters were vacuum packed (FRIMAQ V900, Lorca, Spain) in each polyethylene bag, pasteurized at 90 °C for 30 min and kept at 2 °C storage until the laboratory analysis during the 90-days storage period. Four packages of frankfurters from each batch were taken at 0, 30, 60 and 90 days of storage. All analyses were carried out in triplicate for each formulation. Chemical composition and amino acid profile were evaluated only on day 0. After the sampling for microbial analysis, the sausages were oxygenated for 30 min at room temperature for the rest of analyses.

### 2.3. Chemical Composition

Moisture, ash and protein were quantified according to the International Organisation for Standardisation (ISO) recommended standards 1442:1997 [28], 936:1998 [29] and 937:1978 [30], respectively. Crude fat was extracted using an Ankom XT10 (Ankom Technology Corp., Macedon, NY, USA), according to the American Oil Chemists’ Society (AOCS) Official Procedure Am 5-04 [31]. Protein content was estimated by Kjeldahl method (N × 6.25). Dietary fiber contents (total (TDF), insoluble (IDF) and soluble (SDF) fiber) were determined with the dietary fiber assay kit TDF-100A (Sigma Aldrich, St. Louis, Missouri, U.S.A.) according to the AOAC procedures [32]. The total content of carbohydrate was calculated by difference. Protein and dietary fiber were also measured in the mushroom flours.

Na content was determined from the ashes of 5 g-aliquots of each sample dissolved in 10 mL of 1 M HNO_3_, and analyzed by inductively coupled plasma (ICP)-optical emission spectroscopy (Thermo-Fisher, Cambridge, UK) equipped with a radio frequency source of 27.12 MHz, a peristaltic pump, a spraying chamber and a concentric spray nebuliser, and controlled by the ICP software. Standard solutions (50, 100, 150, 200 mg/L) were prepared from a stock solution of Na (1000 mg/L; SCP-SCIENCE, Courtaboeuf, France) in 4% HNO_3_ (*v*/*v*). The results were expressed as milligrams per 100 g sausage.

### 2.4. Amino Acid Profile

Amino acid composition (g/100 g of sample) of frankfurters was determined using the methodology described by Marti-Quijal et al. [33] by derivatization of amino acids with 6-Aminoquinolyl-N-hydroxysuccinimidyl carbamate (Waters AccQ-Fluor reagent kit) and reversed-phase high-performance liquid chromatography analysis (RP-HPLC) (Waters 2695 Separations Module + Waters 2475 Multi Fluorescence Detector + WatersAccQ-Tag amino acids analysis column). Empower 2 TM advanced software (Waters, Milford, MA, USA) was used to control system operation and results management. Amino acids were identified by retention time using an amino acid standard (Amino Acid Standard H, Thermo, Rockford, IL, USA).

### 2.5. Lipid Oxidation Analysis

The thiobarbituric acid-reactive substances (TBARs) assay was carried to evaluate lipid stability according to the methodology of Vyncke [34]. For this purpose, 2 g of sample and 10 mL of trichloroacetic acid (5%) were homogenised using an Ultra-Turrax (IKA T25 basic, Staufen, Germany) for 2 min. The homogenate was kept at −10 °C for 10 min and centrifuged at 2360 *g* for 10 min. The supernatant was filtered through a Whatman No. 1 (Sigma Aldrich, St. Louis, MO, USA) filter paper. The extract (5 mL) was mixed with a 0.02 M thiobarbituric acid solution (5 mL) and incubated in a water bath at 97 °C for 40 min. The absorbance was measured at 532 nm. For quantification a standard curve of malondialdehyde (MDA) was designed, and the results were expressed as milligrams of MDA per kilogram of sample.

### 2.6. pH and Microbial Analysis

The pH from three frankfurters of each sample was measured with a digital pH-meter (HI 99163, Hanna Instruments, Eibar, Spain) equipped with a glass probe for penetration.

Total viable counts (TVC) and lactic acid bacteria were determined using TEMPO system (TEMPO Filler, TEMPO Reader, BioMérieuxs, Marcy l´Etoile, France), based on the most likely number technology. Both microorganisms were incubated at 30 °C for 24 and 48 h respectively (detection limit of 0.3 CFU/g of sample). *Pseudomonas* spp. counts were enumerated on Pseudomonas agar base with selective supplement for this microbial group (CFC) (Merck, Darmstadt, Germany) after incubation at 25 °C for 48 h. Psychrotrophic aerobic bacteria were enumerated on plate count agar (PCA; Oxoid, Unipath Ltd., Basingstoke, UK) following incubation at 7 °C for 10 days. Detection limits were 2 log CFU/g for pseudomonads and psychrotrophic bacteria. The microbial results were expressed as log CFU/g.

### 2.7. Color and Texture Profile

CIELAB parameters (L*: lightness; a*: redness and b*: yellowness) were measured in slices of 2 cm thickness using a portable colorimeter (Konica Minolta CM-600d, Osaka, Japan) under D65 illuminant and 10° observer with an 8 mm aperture.

A texturometer (TA-XTplus, Stable Micro Systems, Surrey, UK) equipped with Texture Exponent 32 software (version 1.0.0.68) was used to measure the texture profile analysis (TPA). The samples were cut in pieces of 2.5 cm height × 2 cm diameter and hardness (N), springiness (mm), chewiness (N/mm), gumminess (N) and cohesiveness (mm/mm) were determined (Bourne, 1978). Textural parameters were measured by compressing to 50% with a double compression cycle test using an aluminum cylinder probe P50 (50 mm diameter) at speed of 20 mm/s and a distance of 30 mm with a 50 Kg load cell. Three slices of each sample were measured.

### 2.8. Sensory Analysis

Sensory evaluation of frankfurters- type sausage was conducted by twenty trained panelists selected from the members of Meat Technology Center of Galicia, being the participants trained according ISO regulations [35] with the attributes and the scale to evaluate the color, discoloration at surface and odor of raw sausages stored for 0, 30, 60 and 90 days, using a 5-point hedonic scale, ranging from 1 = “excellent” to 5 = “not acceptable” [36]. Also, odor and taste of 1 cm-slices cooked at 180 °C until reach an internal temperature of 72 °C, were evaluated at day 0. Bread without salt and water was used to clean the palate between samples.

### 2.9. Statistical Analysis

Analysis of variance (ANOVA) was performed with Statgraphics Centurion XVI version 16.1.03 (StatPoint Technologies, Inc., Warrenton, VA, USA). Tukey’s test was used to compare the mean values at a significance level of *p* < 0.05.

## 3. Results and Discussion

### 3.1. Chemical Composition

The results of chemical composition of different treatments are shown in Table 2. The addition of mushroom flour resulted in a significant (*p* < 0.05) moisture increase (64.34–66.48%) compared with the control (61.05%). The higher concentration of flour resulted in the highest moisture values in LF-Ab and LF-Po. These moisture increments could be attributed to the higher water amount added to compensate the fat reduction in formulation and the presence of mushroom flour, which is rich in dietary fiber, particularly β-glucans [37]. Contents of total dietary fiber of the mushroom flours Ab and Po were 22.82 ± 0.42% and 43.58 ± 1.33% in dry weight, respectively. Several works have pointed out that the porous and hydrophilic capacity of dietary fiber contributes to water holding properties increasing moisture [38,39]. The increased in moisture has been reported in other fat replacers like konjac gel [40], or pineapple dietary fibers [12].

As expected, batches formulated with lower content of fat and salt resulted in significant (*p* < 0.05) lower concentrations for these parameters due to the modification in the formulations. Fat was reduced from 19.16 ± 0.42 to 16.28 ± 0.33, 15.18 ± 0.29 and 14.04 ± 0.47 in medium fat sausages MF-Ab, MF-Po and MF-PoAb, respectively. In LF-Ab (12.99 ± 0.30) and LF-Po (11.79 ± 0.06) was reached the highest reduction of fat. Na contents of flour added samples were approximately half of the control samples. The same behavior was observed in the ash content, quite related with the sodium chloride content reduction in the formulations [36]. The reduction of fat in meat emulsions can provoke changes in emulsion stability parameters, such as fat and water losses during cooking. Therefore, the meat industry has adopted new trends to improve the texture and water holding capacity, substituting animal fat by including the use of non-meat ingredients, such as inulin or β-glucan, considering the fiber´s ability to retain fat and water [15]. However, when Ab and Po performance are compared, Po was less effective in fat retention, probably related to different protein content of flours, 28.63 ± 0.10% in Ab flour and 16.04 ± 0.22% in Po flour, expressed in dry basis. When the protein content of frankfurters was analyzed, only sausages with 5% Ab mushroom presented a significant higher protein content (*p* < 0.05) despite the higher protein content of Ab flour in agreement with the reported by Reis et al. [41] and Cheung [42]. The expected increase in this parameter because of the mushroom addition was overshadowed by the moisture augmentation reached in mushroom flour added products and the reduction of 50% of sodium caseinate in formulations. When the protein content is reported in dry weight (DW) all mushroom formulations presented significant higher contents (over 40% DW) than the 36.94 DW from control samples, being the LF-Ab, LF-Po the samples with the major values 45.77 g/100 g and 43.52 g/100 g DW, respectively.

The addition of these flours as a source of fiber in meat products has to be highlighted, since fiber content is usually absent in these products. Several works have included different vegetables, legumes or fiber sources to increase the fiber content of these products [11,12]. The batches with addition of the Ab and Po flour showed higher fibre content in a range of 0.57 and 2.18 g/100 g of sausage in comparison with the control (0.05 g/100 g sausage). LF-Po presented the higher value of dietary fibre with 6.51 g/100 g sausage (DW) since the Po flour presented the highest content in dietary fiber. The dietary fiber in mushrooms comes from non-digestible carbohydrates mainly chitin, glucans, cellulose and hemicelluloses like mannans, xylans and galactans [18,42]. With the combination of both mushroom flours in MF-PoAb the reduction of fat is accompanied with an interesting fiber content of 1.58 ± 0.17 being a promising alternative for a healthier sausage keeping the protein content with half of the sodium caseinate added.

### 3.2. Amino Acid Profile

When the amino acid profile of the treatments is analyzed (Table 3) no significant changes were observed between batches (*p* > 0.05). Some studies have been focused on the addition of non-meat protein sources like legumes or even algae in order to improve the protein profile in meat products [10,33]. In this case the addition of mushroom flour did not modify the amino acid profile keeping the ratio between essential and no essential amino acids in 0.93–0.94. The predominant amino acids in formulations were glutamic acid (ranging 2.58–2.84 g/100 g), aspartic acid (1.44–1.66 g/100 g), lysine (1.38–1.54 g/100 g) and leucine (1.31–1.43 g/100 g). These amino acids with alanine and arginine are also the most important in dried edible mushrooms [18], being glutamic and aspartic acids strongly related to umami taste [17]. However, the concentrations of flours added did not change the amino acid profile although sensorially mushroom taste was noticed. Related to essential amino acids lysine, leucine, arginine and valine were the predominant essential amino acids. The edible mushroom has been reported to contain all nine essential amino acids required for human intake [17,43], even though the protein profile of mushrooms depends not only on the specie but also on the size, composition of the substrate and harvest time [18]. In the case of *A. bisporus* and *P. ostreatus* leucine has been reported to be the limiting amino acid but with a protein quality in terms of digestibility and essential amino acid composition comparable to casein, eggs, and soy [17,37]. In this work, 50% of caseinate was substituted by mushroom flour in the formulation, but considering that non significant differences (*p* > 0.05) were found regarding the essential amino acid profiles, mushroom flours could be even suggested to partially replace meat content or to be considered as meat substitute.

### 3.3. Lipid Oxidation Analysis

**Figure 1:**
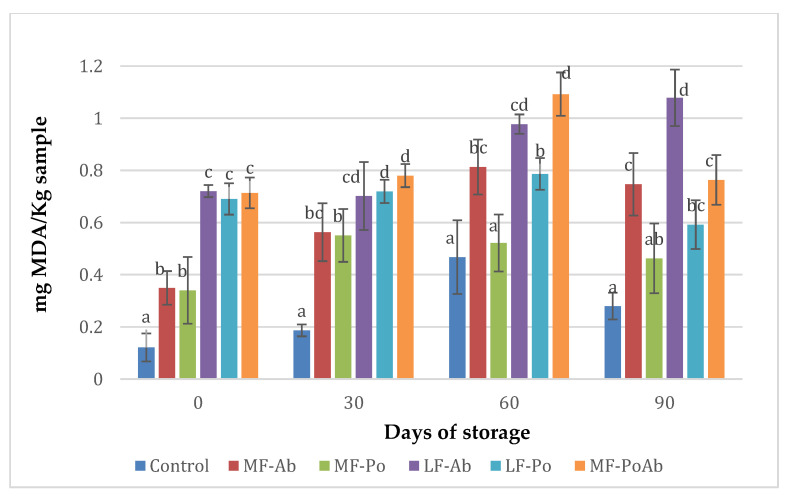
Results of TBARS (mg MDA/Kg) of sausages added with Ab and Po flours. (a–d): Mean values (corresponding to the same day) not followed by a common letter differ significantly (*p* < 0.05).

The oxidative process was evaluated during storage time at 0, 30, 60 and 90 days, and the results are shown in Figure 1. The analysis of variance indicated that TBA values were significantly (*p* < 0.05) affected by the concentration of the edible mushroom flours added and the storage time. Initially, the scores obtained in samples with mushroom flours and less fat were significantly higher (*p* < 0.05) ranging in 0.34–0.72 mg MDA/Kg than the found in control samples with 0.12 mg MDA/Kg. This behaviour remained during cold storage. However, most treatments with Ab and Po flours presented TBA values below the acceptable limit (<1.0 mg MDA/kg) [44]. Mushrooms have been described to possess antioxidant activity, mainly because of the phenolic compounds, although their antioxidant properties depend on the species of mushroom, and the growing, harvest and processing conditions [45,46]. However, the antioxidant effect mushroom flours was not appreciated in this study and the higher concentration of flour (LF-Ab and LF-Po) led to higher initial TBARs, when a reduction of TBARs should be expected, since in these samples a reduction of 50% of fat was applied.

The limited antioxidant activity of mushroom flour and high TBARS found initially in samples with Ab and Po flour could be due to the drying conditions applied to the mushrooms during the obtaining of the flour, which could have promoted browning reaction and protein degradation products, participating in the formation of TBA color complexes and overestimating MDA values, as it has been reported by Papastegriadis et al. [47] in products like dry nuts. Besides, a significant increase was observed during storage for all treatments but after 90 days of cold storage, TBARS values significantly decreased (*p* > 0.05). This behavior was also noticed by Fernandes et al. [48] and attributed to instability or transitory nature of some secondary products from lipid peroxidation like malondialdehyde (MDA). But also, the higher values of TBARs of Ab samples especially from day 60 comparing to Po samples could be attributable to more browning reaction products present in Ab samples reacting with TBA since Ab flour have a darker color.

### 3.4. pH and Microbial Results

**Figure 2:**
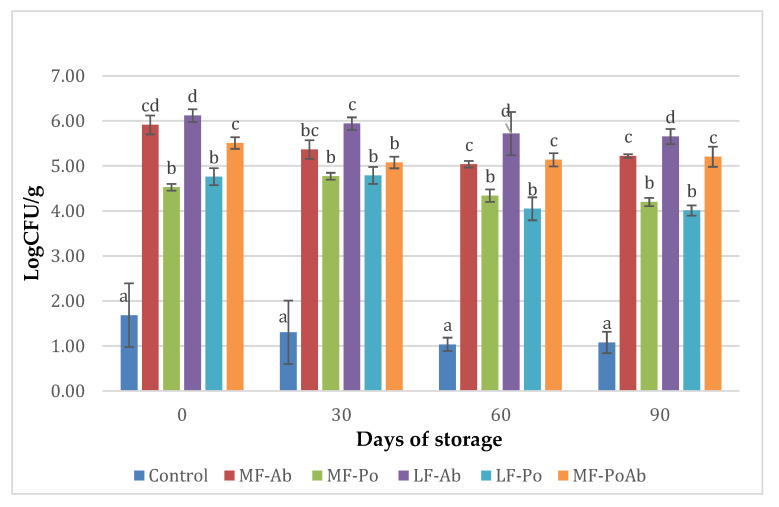
Evolution of microbial counts (Log CFU/g) of sausages during storage with Ab and Po flours. (a–d): Mean values (corresponding to the same day) not followed by a common letter differ significantly (*p* < 0.05).

The inclusion of mushroom flour in the sausage led to a significant increase of pH over 6.0 comparing to 5.94 ± 0.02 Log CFU/g of control samples and the pH values kept during the cold storage. The pH of samples was in the range (5.94–6.11) similar to the pH reported for frankfurters with different levels of shiitake [8], and other cooked sausages with extracts or alternative ingredients added [48]. Microbiologically, the cooking process and post-packing pasteurization eliminated the vegetative microorganisms, so lactic acid bacteria, pseudomonads and psychrotrophic bacteria remained under detection limits. However, counts between 4.52 ± 0.14–6.12 ± 0.08 CFU/g were found in total viable counts for flour mushroom added-frankfurters, much higher than the 1.68 CFU/g reported for control samples, and remained stable during the cold storage (Figure 2). These high levels of microorganisms can be attributed to spore forming bacteria naturally present in agrifoods in contact with soil like vegetables and mushrooms, which survived to the thermal treatment. Ab flour sausages presented significant higher counts (*p* < 0.05) than Po samples, possibly related to the conditioning process, which involved a blanching process, spreading the spore-forming contamination. However, in all cases the high counts are not considered a risk as long as refrigeration temperatures are maintained.

### 3.5. Color and Texture Profile Analysis

**Figure 3:**
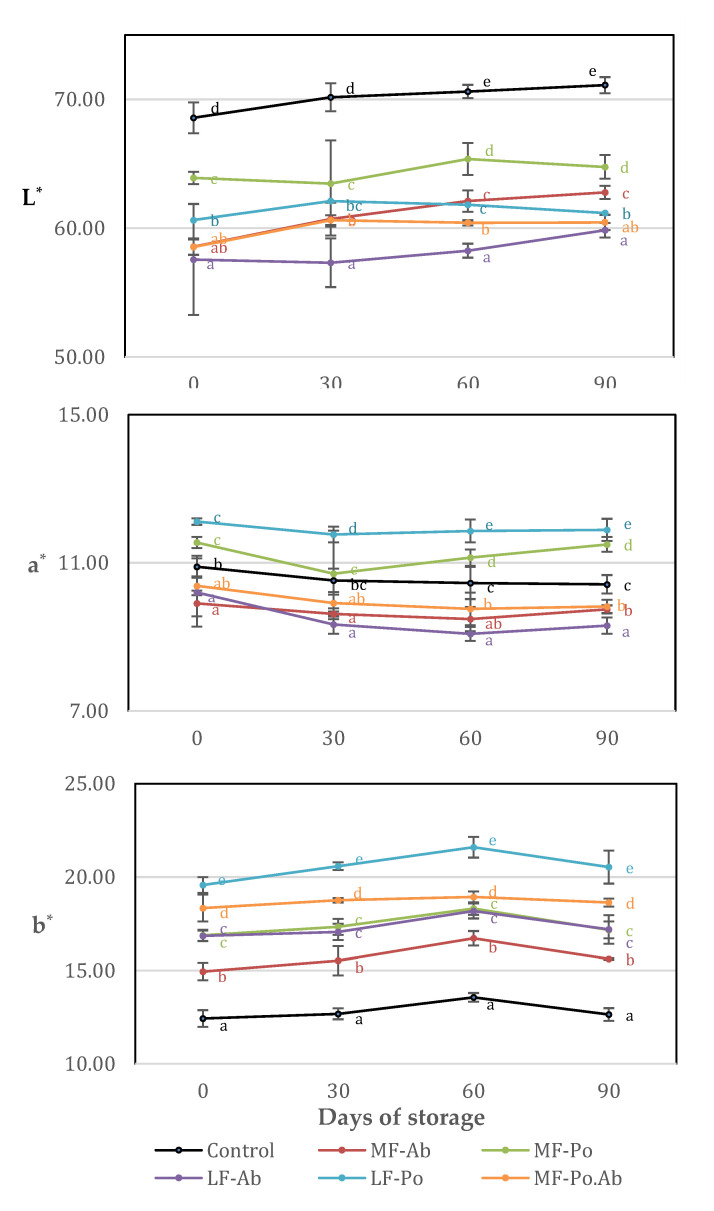
Evolution of color parameters (L*, a* and b*) during the storage of cooked sausages with edible mushroom flours (a–e): Mean values (corresponding to the same day) not followed by a common letter differ significantly (*p* < 0.05).

**Figure 4:**
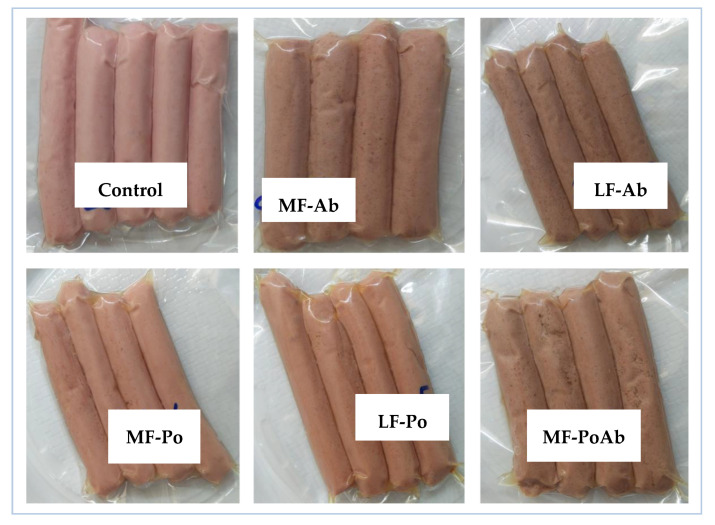
Visual appearance of frankfurter with different formulations.

The color parameters of frankfurters during storage are shown in Figure 3. The addition of mushroom flour significantly reduced (*p* < 0.05) the lightness by the fat reduction procedure, although Po flour decreased the L* parameter to a lesser extend (LF-Po: 60.63 ± 0.35–MF-Po: 63.90 ± 0.48) than Ab flour (LF-Ab: 57.57 ± 4.31–MF-Ab: 58.57 ± 0.64). Yellowness (b*) of the frankfurters increased in flour added frankfurters, especially in samples with Po. Ab samples showed significant lower a* values (*p* < 0.05) comparing with control samples, while addition of Po flour significantly (*p* < 0.05) increased this parameter. So, Ab flour contributed to significantly (*p* < 0.05) reduce the L* and a* values comparing to the control giving a darker color to the frankfurters as can be seen in Figure 4. Visually, the characteristic pink color of the control samples due to nitrosomyoglobine formation was slightly modified into brownish colors. The addition of edible mushroom flours instead of animal fat did not reproduce the color effect of pork backfat on the treated samples, especially in the Ab samples. Fat contributes in emulsions to lighter meat products and usually natural and artificial colorants are added to keep the pink color. The replacement of pork fat from frankfurters or finely comminuted sausages by oleogels [49] have also resulted in lighter and less red meat products. From the brown or greenish color by the incorporation of algae [10,50,51] or chia [11] to the to orange tones by addition of lycopene or carotenoids [52], the change of color will depend not only on the ingredient source, but also on the concentration added [12]. Finally the presence of mushroom flours was not negatively scored in sensory analysis.

Cold storage marginally affected to color parameters as it has been reported with other fat substitutes in frankfurters like chia or vegetal oils in konjac matrix [11,40]. Although natural pigments use to presenting less color stability [52], color frankfurters with mushroom flour remained mostly stable although a slight increase was observed in lightness probably due to the oxidation of fat. No typical discoloration was observed because of the oxidation of myoglobin pigments during storage [11,12].

The addition of 2.5% and 5% of edible mushroom flours also significantly affected (*p* < 0.05) the texture parameters evaluated in frankfurter with partial decrease of fat (30% and 50%) and salt (Table 4). All mushroom added-samples showed significant (*p* < 0.05) lower hardness, springiness, cohesiveness, gumminess and chewiness values comparing to control samples after elaboration. Samples with Po flour (2.5 and 5%; MF-Po and LF-Po), even in combination with Ab flour (MF-PoAb), gave the lowest values in the textural parameters resulting in softer frankfurters. In the emulsions fat is dispersed in small drops in a continuous phase formed by water, proteins and additives. When the fat is reduced the emulsion can loose stability and affect the texture. In general, when the reduction of fat is compensated by increasing protein, textures tend to be harder. However, when fat content is reduced by increasing the proportion of water, keeping the amount of protein, the structure of low-fat systems becomes softer [40]. In this case the replacement of fat and addition of mushroom flour led to a higher water contents in formulation and protein and fibre from mushrooms could help to bound water reducing hardness, and the rest of textural parameters with concentration of flour added. However, differences found according to the origin of mushroom flour, could be related to the different protein content of mushroom flours, since Ab flour (with a higher protein content) reduced to a lesser extend the textural parameters than Po flour (with around 16% of protein but a higher fiber content, over 40%).

Proteins from different sources have been added to meat products like frankfurters to stabilize and compensate the reduction of meat or fat. Stephan et al. [53] reported suitable properties of mycelia from *Pleurotus sapidus* as meat substitute in vegan boiled sausages comparable to the use of other protein concentrates (soy, pea and sunflower), but lower textural parameters than the original german boiled sausage were observed. In this case, the addition of Ab mushroom flour better helped to stabilize the emulsion, even if other protein source like sodium caseinate was reduced in the formulation.

In general, softer textures have been obtained when different vegetal, cereal or legume, algae or fiber sources have been included in meat formulations with or without replacement of fat or other ingredients. Alvarez et al. [15] found a significant decrease of hardness in frankfurters by addition of rice bran and walnut. Choi et al. [44] reported a significant decrease of hardness in reduced- fat frankfurters with vegetable oils and rice bran fiber, and Cofrades et al. [54] observed that increasing amounts of walnut extracts reduced shear force and elongation values, indicating the formation of softer and less cohesive meat structures. Addition of 1% of pineapple fiber in partial replacement of fat in sausages significantly reduced hardness, chewiness and gumminess but less differences were appreciated in cohesiveness and springiness [12]. The partial replacement of fat and salt by addition of 1% of different seaweeds resulted also in lower hardness and chewiness parameters although the behavior on adhesiveness and springiness was seaweed-species related [50]. The addition of concentration of 0.8–1.2% of shiitake (*Lentinus edodes*) powder in frankfurter also reduced hardness, but increased cohesiveness [8].

In general, texture parameters were also affected (*p* < 0.05) by cold storage increasing all the parameters, but especially hardness in control samples comparing with mushroom added sausages. An increase of hardness during storage has been reported in frankfurters as a consequence to the increase of purge loss [40].

### 3.6. Sensory Evaluation

**Figure 5:**
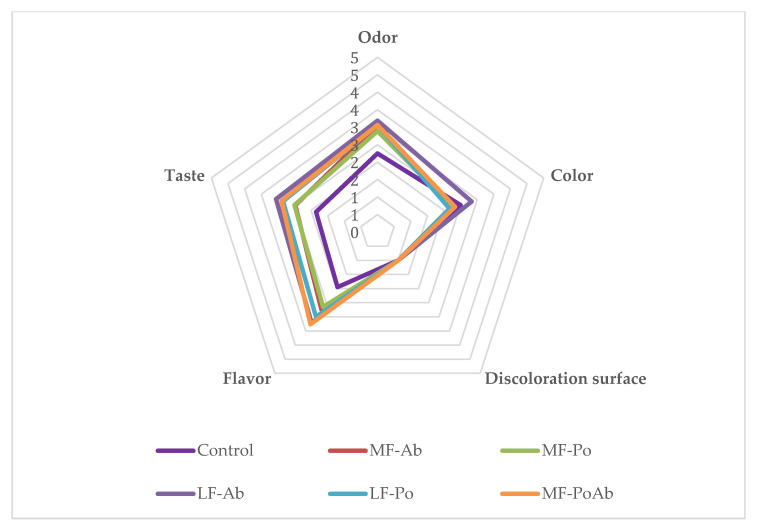
Sensory results of frankfurters evaluated at day 0.

In the case of sensory attributes evaluated in samples before cooking (color, decoloration surface), no significant differences (*p* > 0.05) were detected between treatments with color values between good and acceptable (2.17–2.83) and no discoloration was observed (Figure 5). The evaluation of odor indicated that samples with Ab (MF-Ab, LF-Ab, MF-PoAb) and LF-Po presented a stronger mushroom odor, with scores over acceptable level, while MF-Po did not significantly (*p* > 0.05) differ from control. When the samples were cooked the reduction of fat and salt and the inclusion of Ab and Po flours resulted in lower sensorial values for flavor and taste but around acceptability level, without significant differences between flour added samples. In the case of flavor, the samples with 5% of flour (LF-Ab, LF-Po and MF-PoAb) scored over acceptable level (3.0) while control samples scored near good level (1.95 ± 0.76). In the taste parameter, only LF-Ab scored over acceptable level, while de rest of treatments were rated between good and acceptable levels. The characteristic flavor or umami in mushrooms is intense, especially in Ab samples [20], and the acceptability of the frankfurter will depend on how accustomed the consumer to this flavor. When aqueous extract from other edible mushroom, *Cantharella cibarius*, was added to frankfurter, it was not sensorially different from control samples [26]. In general, umami compounds enhance palatability in savory foods which allows to reduce salt content without a negatively perception in the consumer increasing also satiety [55]. But the incorporation of new ingredients into processed meats modifies sensory properties and can limit the consumer acceptability [10]. However, this untypical or unexpected flavor could be moderated by incorporating different seasonings, since no flavor additives were included in formulations here tested, and even control samples were scored as good, but not excellent, because of the plain flavor. Jimenez-Colmenero et al. [51] showed that the addition of konjac-seaweed flour to low fat and low salt frankfurters slightly reduced sensory panel values, due to an intense unfamiliar flavor. However, they did not consider this an obstacle, and suggested the reformulation using less strongly flavored seaweed and considering seasonings.

During cold storage, sensory parameters evaluated in uncooked samples, color, discoloration and odor did not significantly changed confirming the stability of the product during long periods of storage.

## 4. Conclusions

The inclusion of Ab and Po flours in fat- and salt-reduced frankfurter sausages seems to a feasible strategy to enhance the nutritional profile these products, although the physicochemical, textural and sensory properties were affected by the mushroom and concentration source. The protein profile did not changed, but fiber contents were improved, which makes Ab and Po flour an interesting substitute for fat and salt and even for meat. Color was significantly affected, especially by the inclusion of Ab flour, resulting in darker products, while Po flour addition presented a higher impact in the texture with softer and less cohesive sausages. Nevertheless, all mushroom flours remained sensorially acceptable despite the strong umami flavor was perceived. During cold storage, samples remained quite unchanged for 90 days.

## Figures and Tables

**Table 1:** Formulation (g) of different frankfurters with edible mushroom flour.

	Control ^1^	MF-Ab	MF-Po	LF-Ab	LF-Po	MF-PoAb
Lean meat	2500	2500	2500	2500	2500	2500
Flour *Agaricus bisporus*	0	125	0	250	0	125
Flour *Pleurotus ostreatus*	0	0	125	0	250	125
Pork backfat	1250	875	875	625	625	875
Sodium caseinate	100	50	50	50	50	50
Sodium chloride	75	37.5	37.5	37.5	37.5	37.5
Water	1000	1346	1346	1471	1471	1471
Potato starch	50	50	50	50	50	50
Di-tri phosphates	25	12.5	12.5	12.5	12.5	12.5
Sodium nitrite	1.5	1.5	1.5	1.5	1.5	1.5
Sodium ascorbate	2.5	2.5	2.5	2.5	2.5	2.5

^1^ Batches: Control (25% fat, 1.5% salt, 2% Sodium caseinate and 0.5% phosphates), MF-Ab (30% fat reduction, 50% salt reduction, 2.5% Ab), MF-Po (30% fat reduction, 50% salt reduction, 2.5% Po), LF-Ab (50% fat reduction, 50% salt reduction, 5.0% Ab), LF-Po (50% fat reduction, 50% salt reduction, 5.0% Po) and MF-PoAb (30% fat reduction, 50% salt reduction, 2.5% Ab and 2.5% Po).

**Table 2:** Proximate composition (%) and content sodium (mg/100 g) of frankfurters type sausage elaborated with flour of edible mushroom (*Agaricus bisporus* and *Pleurotus ostreatus*).

	Control	MF-Ab	MF-Po	LF-Ab	LF-Po	MF-PoAb
Moisture	61.05 ± 0.17 ^a^	64.34 ± 0.13 ^b^	65.91 ± 0.21 ^d^	66.34 ± 0.50 ^de^	66.48 ± 0.30 ^e^	65.37 ± 0.55 ^c^
Fat	19.16 ± 0.42 ^f^	16.28 ± 0.33 ^e^	15.18 ± 0.29 ^d^	12.99 ± 0.30 ^b^	11.79 ± 0.06 ^a^	14.04 ± 0.47^c^
Protein	14.39 ± 0.14 ^b^	14.70 ± 0.27 ^b^	13.62 ± 0.22 ^a^	15.41 ± 0.54 ^c^	14.59 ± 0.33 ^b^	14.29 ± 0.24 ^b^
Ash	2.08 ± 0.05 ^d^	1.30 ± 0.04 ^a^	1.36 ± 0.06 ^ab^	1.36 ± 0.08 ^ab^	1.81 ± 0.03 ^c^	1.41 ± 0.05 ^b^
Carbohydrates	3.32 ± 0.25 ^a^	3.37 ± 0.39 ^a^	3.93 ± 0.31 ^b^	3.90 ± 0.19 ^b^	5.33 ± 0.04 ^d^	4.88 ± 0.21 ^c^
Dietary fiber	0.05 ± 0.09 ^a^	0.57 ± 0.06 ^b^	1.09 ± 0.11 ^c^	1.14 ± 0.09 ^c^	2.18 ± 0.25 ^e^	1.58 ± 0.17 ^d^
Na (mg/100g)	686.30 ± 37.10 ^c^	338.69 ± 40.53 ^a^	336.46 ± 17.97 ^a^	345.22 ± 30.47 ^a^	405.19 ± 12.53 ^b^	347.48 ± 4.07 ^a^

Results are expressed as mean value ± standard deviation. ^a–f^: Different letters in each batch indicate significant differences (*p* < 0.05).

**Table 3:** Amino acid profile (g/100 g sample) of protein from frankfurters with addition of edible mushroom flours.

	Control	MF-Ab	MF-Po	LF-Ab	LF-Po	MF-PoAb
OH-Proline	0.21 ± 0.01	0.18 ± 0.01	0.15 ± 0.01	0.18 ± 0.01	0.18 ± 0.06	0.19 ± 0.02
Aspactic acid	1.44 ± 0.20	1.52 ± 0.03	1.49 ± 0.01	1.66 ± 0.20	1.44 ± 0.29	1.62 ± 0.19
Serine	0.65 ± 0.07	0.64 ± 0.03	0.64 ± 0.01	0.72 ± 0.06	0.65 ± 0.11	0.73 ± 0.03
Glutamic acid	2.67 ± 0.37	2.65 ± 0.01	2.59 ± 0.01	2.81 ± 0.29	2.58 ± 0.55	2.84 ± 0.26
Glycine	0.95 ± 0.06	0.85 ± 0.01	0.92 ± 0.01	0.95 ± 0.05	1.00 ± 0.21	1.01 ± 0.01
Alanine	0.88 ± 0.10	0.88 ± 0.01	0.91 ± 0.03	0.93 ± 0.10	0.90 ± 0.23	0.95 ± 0.08
Cysteine	0.10 ± 0.01	0.09 ± 0.01	0.09 ± 0.02	0.11 ± 0.01	0.11 ± 0.02	0.12 ± 0.01
* Valine	0.91 ± 0.14	0.89 ± 0.04	0.90 ± 0.06	0.96 ± 0.12	0.92 ± 0.24	0.98 ± 0.04
Methionine	0.40 ± 0.05	0.40 ± 0.01	0.38 ± 0.01	0.41 ± 0.10	0.42 ± 0.09	0.48 ± 0.06
* Isoleucine	0.81 ± 0.15	0.80 ± 0.05	0.79 ± 0.04	0.85 ± 0.12	0.79 ± 0.19	0.87 ± 0.04
* Leucine	1.35 ± 0.24	1.33 ± 0.09	1.31 ± 0.08	1.41 ± 0.19	1.31 ± 0.32	1.43 ± 0.06
Tyrosine	0.40 ± 0.05	0.40 ± 0.02	0.38 ± 0.04	0.46 ± 0.04	0.42 ± 0.07	0.44 ± 0.03
* Phenylalanine	0.69 ± 0.10	0.67 ± 0.06	0.67 ± 0.04	0.73 ± 0.08	0.69 ± 0.13	0.73 ± 0.01
* Hystidine	0.54 ± 0.03	0.51 ± 0.01	0.52 ± 0.01	0.57 ± 0.06	0.56 ± 0.11	0.58 ± 0.01
* Lysine	1.45 ± 0.20	1.50 ± 0.04	1.46 ± 0.01	1.54 ± 0.12	1.38 ± 0.28	1.53 ± 0.22
* Arginine	0.93 ± 0.09	0.92 ± 0.03	0.90 ± 0.01	0.97 ± 0.05	0.90 ± 0.14	0.97 ± 0.12
Proline	1.05 ± 0.17	0.97 ± 0.04	0.96 ± 0.07	1.02 ± 0.13	0.94 ± 0.34	1.05 ± 0.02
Taurine	<0.01	<0.01	<0.01	<0.01	<0.01	<0.01
* Threonine	0.73 ± 0.08	0.73 ± 0.03	0.71 ± 0.02	0.78 ± 0.04	0.72 ± 0.12	0.80 ± 0.07
Total	16.16 ± 2.09	15.94 ± 0.39	15.79 ± 0.29	17.07 ± 1.77	15.93 ± 3.51	17.35 ± 1.06
Essential (E)	7.81 ± 1.09	7.74 ± 0.27	7.64 ± 0.20	8.22 ± 0.88	7.70 ± 1.63	8.38 ± 0.51
Non-essential (Ne)	8.35 ± 1.00	8.19 ± 0.12	8.14 ± 0.09	8.85 ± 0.89	8.23 ± 1.88	8.97 ± 0.55
E/Ne	0.93 ± 0.02	0.94 ± 0.02	0.94 ± 0.01	0.93 ± 0.01	0.94 ± 0.02	0.93 ± 0.01

*: Essential amino acid.

**Table 4:** Texture parameters of frankfurters with edible mushroom flours.

Texture Parameter	Days of Storage			
	0	30	60	90
**Hardness (N)**				
Control	20.56 ± 1.01 ^d,X^	25.12 ± 2.79 ^c,Y^	24.18 ± 0.06 ^d,XY^	27.84 ± 3.90 ^c,Y^
MF-Ab	15.33 ± 2.63 ^c,X^	15.15 ± 0.94 ^ab,X^	16.33 ± 1.83 ^c,X^	17.46 ± 1.46 ^b,X^
MF-Po	13.53 ± 2.26 ^bc,X^	16.83 ± 1.69 ^b,Y^	16.40 ± 2.15 ^c,XY^	14.75 ± 1.98 ^ab,XY^
LF-Ab	12.07 ± 1.25 ^ab,X^	16.42 ± 1.17 ^b,Y^	13.67 ± 1.83 ^ab,X^	16.09 ± 1.05 ^b,Y^
LF-Po	10.84 ± 0.44 ^a,X^	13.41 ± 1.62 ^a,Y^	12.52 ± 1.40 ^a,XY^	13.09 ± 0.69 ^ab,Y^
MF-PoAb	11.50 ± 0.95 ^ab,X^	13.02 ± 0.66 ^a,X^	16.08 ± 1.68 ^bc,Y^	14.93 ± 1.25 ^ab,Y^
**Springiness (mm)**				
Control	0.78 ± 0.03 ^c,X^	0.82 ± 0.01 ^c,Y^	0.81 ± 0.01 ^e,Y^	0.81 ± 0.01 ^c,Y^
MF-Ab	0.70 ± 0.04 ^b,X^	0.73 ± 0.04 ^b,XY^	0.75 ± 0.02 ^d,Y^	0.74 ± 0.03 ^bc,XY^
MF-Po	0.59 ± 0.04 ^a,X^	0.65 ± 0.05 ^a,X^	0.64 ± 0.05 ^ab,X^	0.63 ± 0.03 ^a,X^
LF-A	0.66 ± 0.02 ^b,X^	0.74 ± 0.02 ^b,Y^	0.71 ± 0.03 ^cd,Y^	0.75 ± 0.02 ^c,Y^
LF-Po	0.55 ± 0.02 ^a,X^	0.68 ± 0.08 ^ab,Y^	0.62 ± 0.04 ^a,XY^	0.69 ± 0.05 ^b,Y^
MF-PoAb	0.59 ± 0.03 ^a,X^	0.65 ± 0.05 ^a,XY^	0.69 ± 0.03 ^bc,Y^	0.70 ± 0.05 ^bc,Y^
**Cohesiveness (mm/mm)**				
Control	0.38 ± 0.01 ^c,X^	0.44 ± 0.06 ^c,Y^	0.42 ± 0.02 ^d,XY^	0.39 ± 0.01 ^e,XY^
MF-Ab	0.34 ± 0.01 ^bc,X^	0.34 ± 0.01 ^ab,X^	0.36 ± 0.01 ^c,X^	0.36 ± 0.02 ^cd,X^
MF-Po	0.29 ± 0.03 ^a,X^	0.31 ± 0.03 ^a,X^	0.31 ± 0.01 ^a,X^	0.29 ± 0.02 ^a,X^
LF-Ab	0.34 ± 0.01 ^bc,X^	0.37 ± 0.01 ^b,YZ^	0.34 ± 0.02 ^bc,XY^	0.38 ± 0.02 ^de,Z^
LF-Po	0.31 ± 0.02 ^ab.X^	0.34 ± 0.02 ^ab,Y^	0.33 ± 0.01 ^ab,XY^	0.33 ± 0.02 ^b,XY^
MF-PoAb	0.31 ± 0.03 ^ab,X^	0.32 ± 0.01 ^a,X^	0.33 ± 0.02 ^ab,X^	0.34 ± 0.01 ^bc,X^
**Gumminess (N)**				
Control	7.73 ± 0.32 ^c,X^	10.79 ± 1.44 ^c,Y^	10.06 ± 0.30 ^d,Y^	10.81 ± 1.53 ^d,Y^
MF-Ab	5.24 ± 1.27 ^b,X^	5.14 ± 0.45 ^ab,X^	5.86 ± 0.78 ^c,X^	6.29 ± 0.63 ^c,X^
MF-Po	3.99 ± 1.03 ^a,X^	5.22 ± 0.74 ^ab,Y^	5.11 ± 0.52 ^abc,XY^	4.34 ± 0.80 ^a,XY^
LF-Ab	4.10 ± 0.53 ^ab,X^	6.00 ± 0.27 ^b,Y^	4.73 ± 0.84 ^ab,X^	6.04 ± 0.46 ^bc,Y^
LF-Po	3.39 ± 0.26 ^a,X^	4.59 ± 0.72 ^a,Y^	4.13 ± 0.47 ^a,Y^	4.30 ± 0.20 ^a,Y^
MF-PoAb	3.66 ± 0.71 ^a,X^	4.18 ± 0.45 ^a,XY^	5.32 ± 0.88 ^bc,Z^	5.05 ± 0.34 ^ab,YZ^
**Chewiness (N·mm)**				
Control	6.01 ± 0.46 ^c,X^	8.80 ± 1.09 ^d,Y^	8.21 ± 0.13 ^d,Y^	8.82 ± 1.17 ^c,Y^
MF-Ab	3.67 ± 0.98 ^b,X^	3.77 ± 0.29 ^bc,X^	4.41 ± 0.75 ^c,X^	4.65 ± 0.61 ^b,X^
MF-Po	2.37 ± 0.75 ^a,X^	3.41 ± 0.75 ^ab,Y^	3.31 ± 0.59 ^ab,XY^	2.71 ± 0.42 ^a,XY^
LF-Ab	2.70 ± 0.34 ^a,X^	4.46 ± 0.31 ^c,Y^	3.40 ± 0.73 ^ab,X^	4.52 ± 0.48 ^b,Y^
LF-Po	1.87 ± 0.20 ^a,X^	3.15 ± 0.82 ^ab,Y^	2.58 ± 0.41 ^a,XY^	2.98 ± 0.33 ^a,Y^
MF-PoAb	2.17 ± 0.51 ^a,X^	2.74 ± 0.45 ^a,XY^	3.70 ± 0.80 ^bc,Z^	3.56 ± 0.39 ^a,YZ^

Results are expressed as mean value ± standard deviation. (X–Z): Means in the same row not followed by a common letter are significantly different (*p* < 0.05). (a–e): Mean values in the same column (for each texture parameter) not followed by a common letter are significantly different (*p* < 0.05).
